# Knockdown of mechanosensitive adaptor Hic-5 ameliorates post-traumatic osteoarthritis in rats through repression of MMP-13

**DOI:** 10.1038/s41598-023-34659-x

**Published:** 2023-05-08

**Authors:** Aya Miyauchi, Masahito Noguchi, Xiao-Feng Lei, Masashi Sakaki, Momoko Kobayashi-Tanabe, Shogo Haraguchi, Akira Miyazaki, Joo-ri Kim-Kaneyama

**Affiliations:** 1 Department of Biochemistry, Showa University School of Medicine, 1-5-8 Hatanodai, Shinagawa-ku, Tokyo, 142-8555 Japan; 2 Department of Medicine, Division of Gastroenterology, Showa University School of Medicine, 1-5-8 Hatanodai, Shinagawa-ku Tokyo, 142-8666, Japan

**Keywords:** Osteoarthritis, Mechanisms of disease, Focal adhesion, Mechanotransduction

## Abstract

Osteoarthritis (OA) is the most common joint disease associated with articular cartilage destruction. Matrix metalloproteinase-13 (MMP-13) has an essential role in OA pathogenesis by degradation of collagen II, a major component of articular cartilage. Hydrogen peroxide-inducible clone-5 (Hic-5; TGFB1I1), a transforming growth factor-β-inducible mechanosensor, has previously been reported to promote OA pathogenesis by upregulating MMP-13 expression in mouse osteoarthritic lesions. In our current study, immunohistochemical analysis showed that Hic-5 protein expression was increased in human OA cartilage compared with normal cartilage. Functional experiments demonstrated that Hic-5 and MMP-13 expression was increased by mechanical stress, and mechanical stress-induced MMP-13 expression was suppressed by Hic-5 siRNA in human chondrocytes. Moreover, intracellular localization of Hic-5 shifted to the nucleus from focal adhesions in human chondrocytes subjected to mechanical stress, and nuclear Hic-5 increased MMP-13 gene expression. In vivo, intra-articular injection of Hic-5 siRNA decreased the Osteoarthritis Research Society International score and MMP-13 protein expression in articular cartilage of OA rats. Our findings suggest that Hic-5 regulates transcription of MMP-13 in human chondrocytes, and Hic-5 may be a novel therapeutic target for OA because OA progression was suppressed by intra-articular injection of Hic-5 siRNA in rats.

## Introduction

Osteoarthritis (OA) is the most prevalent joint disorder characterized by articular cartilage degradation. A large number of studies have revealed that multiple risk factors contribute to OA development, including obesity, genetic predisposition, aging, trauma, inflammation, and excessive mechanical stress^1–4^. Previous in vitro experiments using cell-stretcher systems have shown that excessive mechanical stress induces expression of matrix metalloproteinase-13 (MMP-13) that has an essential role in OA pathogenesis by degradation of collagen II, a major component of articular cartilage^5–7^. MMP-13 knockout in mice protects against cartilage degradation caused by surgical induction compared with wildtype (WT) mice^8^. Conversely, transgenic mice with constitutively active MMP-13 expressed specifically in cartilage show articular cartilage erosion similar to human OA^9^.

Hydrogen peroxide-inducible clone-5 (Hic-5) is a scaffold protein isolated as a gene induced by hydrogen peroxide and transforming growth factor β (TGF-β)^10^. We have previously demonstrated that subcellular localization of Hic-5 shifts from focal adhesions to stress actin fibers in response to mechanical stress and Hic-5 controls the contractile capability of the cell^11^. Furthermore, Hic-5 has been reported to be involved in the pathogenesis of various disorders. Our previous study showed that Hic-5 increases activated MMP-2 by regulating the expression of membrane type-1 MMP, which leads to the formation of abdominal aortic aneurysms and rupture^12^. In breast tumors, Hic-5 is required for extracellular matrix (ECM) deposition and cell contractility, and metastasis to the lungs decreases in Hic-5-deficient mice^13^. Although there are differences in the detailed mechanisms of Hic-5 involvement in these disorders, ECM remodeling is a common mechanism.

Recently, we found that Hic-5 expression increases in mouse articular cartilage during early OA development, and mice lacking Hic-5 have significantly less cartilage erosion than WT mice^14^. In vitro experiments using murine chondrocytes also demonstrated that Hic-5 deficiency decreases MMP-13 expression induced by excessive mechanical stress. This study aimed to determine whether Hic-5 is involved in MMP-13 expression induced by excessive mechanical stress in human chondrocyte, and to investigate the efficacy of Hic-5 as a therapeutic target for OA in vivo.

## Results

### Significant increase in Hic-5 expression in human OA cartilage

**Figure 1:**
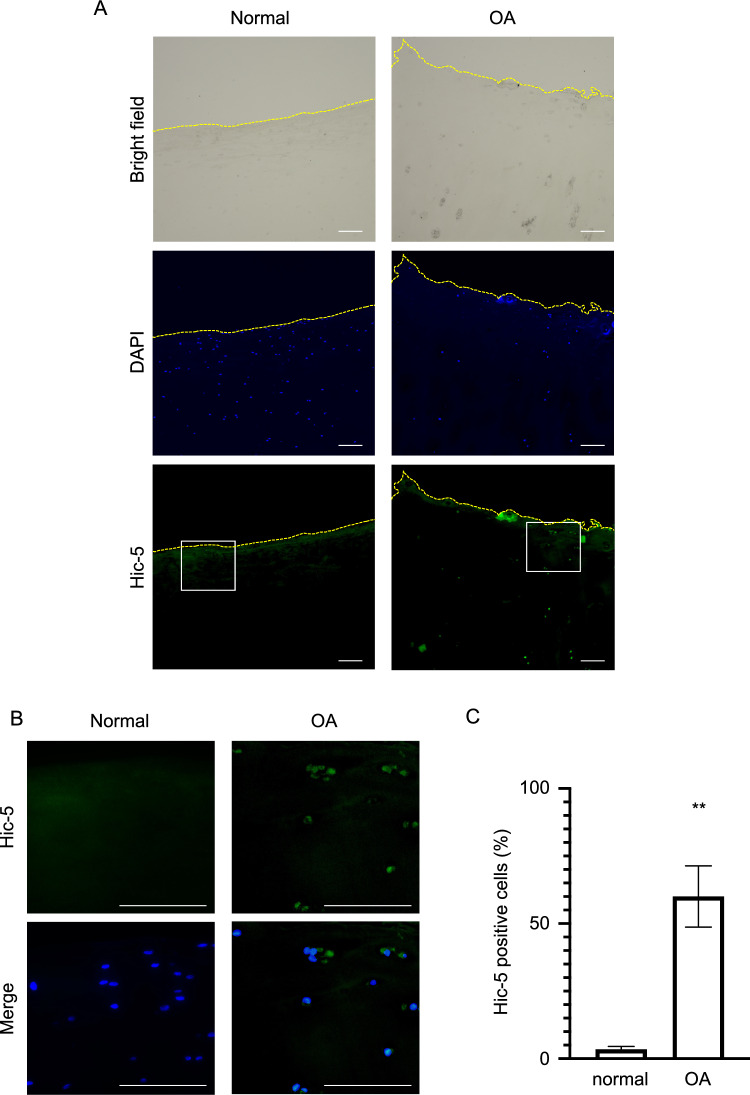
Induction of hydrogen peroxide-inducible clone-5 (Hic-5) expression in human osteoarthritis (OA) cartilage. (**A**,**B**) Representative immunohistochemical staining of Hic-5 in normal and OA cartilage. Broken lines indicate the articular cartilage surface. Original magnification × 100 in (**A**); × 400 in (**B**). Bar = 100 μm. (**C**) Quantification of Hic-5-positive cells in normal (n = 3) and OA cartilage (n = 3). Data were analyzed by the unpaired *t* test. Values are the mean ± SEM. **P < 0.01.

To explore the role of Hic-5 in human OA pathogenesis, we first examined Hic-5 expression in human OA cartilage. Immunohistochemistry showed that Hic-5 expression was higher in OA cartilage than in normal cartilage (Fig. 1A,B), and the number of Hic-5-positive cells was significantly increased in OA cartilage (Fig. 1C). These results were consistent with our previous report indicating that Hic-5 was highly expressed in the cartilage of mice with surgically induced OA^14^.

### Suppression of MMP-13 expression induced by mechanical stress following Hic-5 knockdown in human chondrocytes

**Figure 2:**
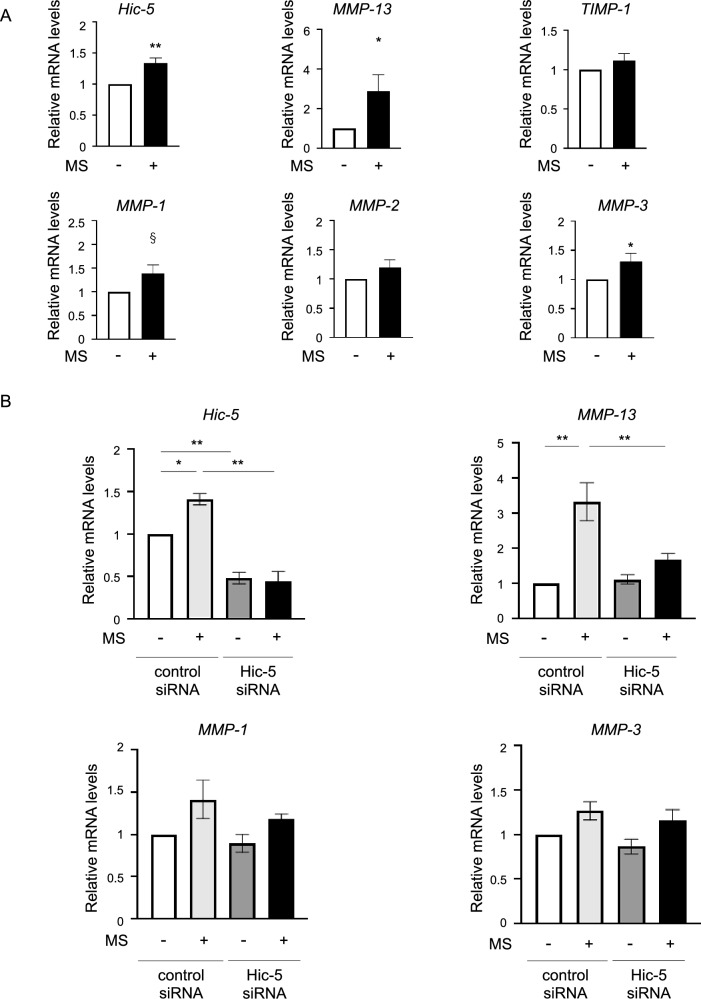
Attenuation of mechanical stress induced-matrix metalloproteinase (MMP-13) expression by Hic-5 knockdown in human chondrocytes. (**A**) mRNA levels of *Hic-5*, *MMPs*, and tissue inhibitor of matrix metalloproteinase-1 (*TIMP-1*) in human chondrocytes exposed to mechanical stress (MS+) for 30 min or untreated (MS−). Cells were collected at 1 h after mechanical stress. (n = 8 biological replicates). (**B**) Changes in gene expression in response to Hic-5 knockdown in human chondrocytes with or without mechanical stress. Human chondrocytes were treated with Hic-5 siRNA (10 nM) or control siRNA (10 nM) for 24 h before stimulation by mechanical stress. (n = 4 biological replicates). Relative levels of mRNA were determined by quantitative reverse transcription-polymerase chain reaction. Values are the mean ± SEM. *P < 0.05; **P < 0.01; ^§^*P* = 0.0572 by the unpaired *t* test in (**A**) or one-way analysis of variance with Tukey’s test for multiple comparisons in (**B**).

In the previous study, we demonstrated that Hic-5 and MMP-13 expression in mouse articular chondrocytes was increased by excessive mechanical stress^14^. Therefore, we investigated whether human articular chondrocytes also had increased expression of *Hic-5* and *MMP-13* induced by mechanical stress in addition to other MMPs and tissue inhibitor of matrix metalloproteinase-1 (*TIMP-1*). As a result, *Hic-5, MMP-3*, and *MMP-13* mRNA levels were increased in human chondrocytes stimulated by mechanical stress compared with unstimulated chondrocytes (Fig. 2A). *MMP-1* expression tended to be upregulated by mechanical stress, but the difference was not significant, and there was no effect of mechanical stress on *MMP-2* or *TIMP-1* expression. Next, we examined the effect of Hic-5 knockdown on MMP gene expression using Hic-5 small interfering RNA (siRNA) and control siRNA as negative control. Although Hic-5 knockdown by siRNA did not alter *MMP-13* expression without mechanical stress, mechanical stress-induced *MMP-13* expression was significantly reduced by Hic-5 siRNA compared with control siRNA. However, Hic-5 siRNA had no effect on *MMP-1* or *MMP-3* expression (Fig. 2B). These results were similar to our previous results observed in chondrocytes isolated from Hic-5 knockout mice, indicating that Hic-5 specifically regulated MMP-13 gene expression among other MMPs under mechanical stress.

### Translocation of Hic-5 from focal adhesions to the nucleus in response to mechanical stress

**Figure 3:**
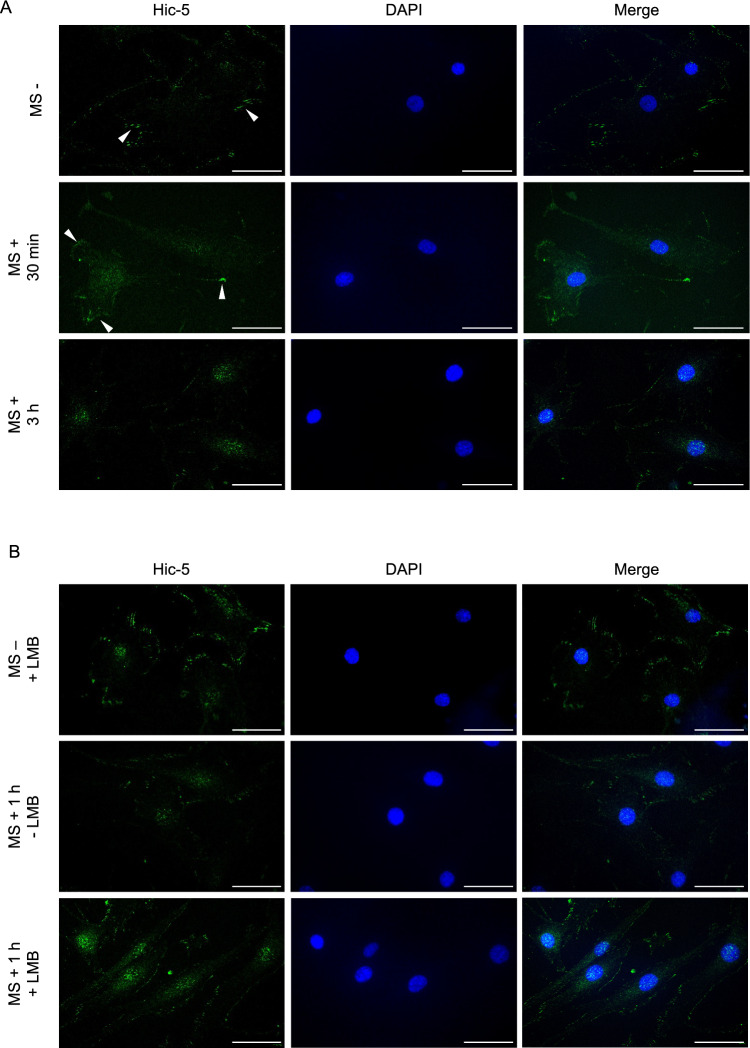
Subcellular localization of Hic-5 in human chondrocytes stimulated by mechanical stress. (**A**) Intracellular localization of Hic-5 in human chondrocytes exposed to mechanical stress (MS+) for 30 min or 3 h, or untreated (MS−). Arrow heads indicate focal adhesions. (**B**) Immunofluorescence imaging of nuclear Hic-5 in human chondrocytes after mechanical stress. Human chondrocytes were exposed to 10 ng/ml leptomycin B (LMB) and mechanical stress for 1 h. Nuclei were counterstained with DAPI (blue). Representative image was selected from 3 biological replicates. Original magnification: × 400. Bar = 50 μm.

We next observed intracellular localization of Hic-5 in human chondrocytes with or without mechanical stress. Hic-5 was detected at focal adhesions under unstimulated conditions, whereas Hic-5 expression in focal adhesions was attenuated after 0.5 Hz, 10% cyclic tensile strain loading for 30 min (Fig. 3A). Moreover, Hic-5 staining was prominent in the nucleus after cyclic tensile strain loading for 3 h (Fig. 3A). Hic-5 normally shuttles between focal adhesions and the nucleus via a nuclear export signal (NES)^15^. Considering the possibility that mechanical stress accelerates the shuttle velocity, we examined changes in the subcellular localization of Hic-5 after cyclic tensile strain under treatment with leptomycin B (LMB), an NES inhibitor. The signal intensity in the nucleus was slightly increased by LMB at 1 h after treatment, whereas the addition of both mechanical stress for 1 h and LMB clearly induced nuclear localization of Hic-5 in human chondrocytes (Fig. 3B). Our previous study reported that nucleus-accumulated Hic-5 in response to H_2_O_2_ participates in transcriptional control of c-*fos*^15^. Taken together, these data imply that translocation of Hic-5 from focal adhesions to nucleus caused by mechanical force regulated MMP-13 expression.

### Induction of MMP-13 expression by nuclear Hic-5

**Figure 4:**
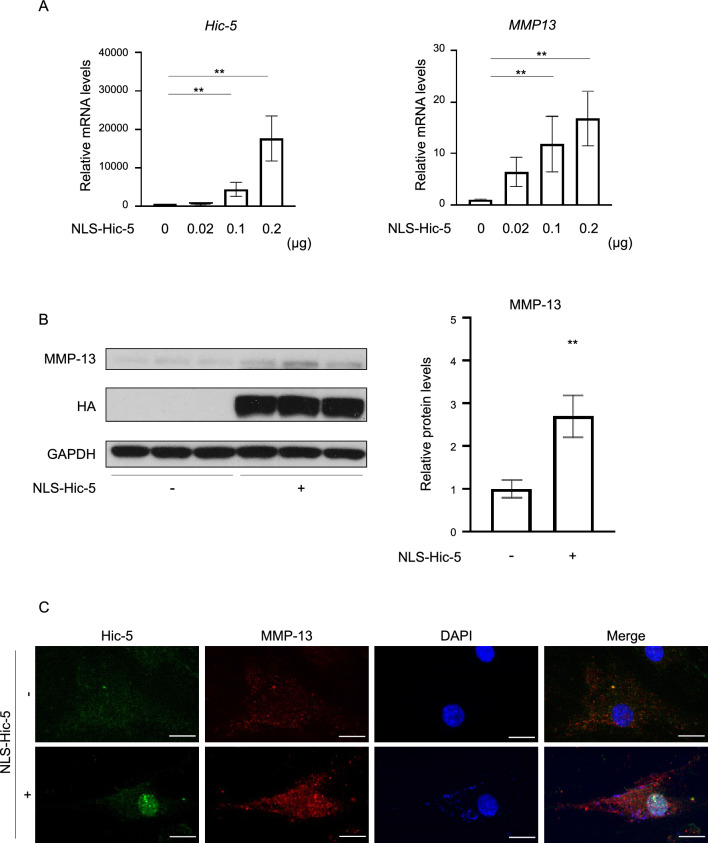
Upregulation of MMP-13 expression by nuclear Hic-5 in human chondrocytes. (**A**) Induction of *MMP-13* in human chondrocytes exogenously expressing Hic-5 tagged with a nuclear localization signal (NLS-HA-Hic-5). Human chondrocytes were transfected with the NLS-HA-Hic-5 expression vector at the concentrations shown in the graph for 24 h. Hic-5 and MMP-13 expression was measured by quantitative polymerase chain reaction (n = 3 biological replicates). (**B**) Western blot of MMP-13 in human chondrocytes transfected with or without 0.2 µg of NLS-Hic-5 (n = 3 biological replicates). Values are the mean ± SEM. **P < 0.01 by the Kruskal–Wallis test, followed by Dunn’s multiple comparisons test in (**A**) or the unpaired *t* test in (**B**). Western blotting images were cropped, and full-length blots are included in Supplementary Fig. 1. (**C**) Double immunofluorescence staining of Hic-5 (green) and MMP-13 (red) in human chondrocytes transfected with or without NLS-HA-Hic-5. Nuclei were counterstained with DAPI (blue). Representative image was selected from 3 biological replicates. Original magnification: × 400. Bar = 50 μm.

To determine whether MMP-13 expression was induced by nucleus-localized Hic-5 in human chondrocytes, we forcibly expressed Hic-5 in the nucleus using a nuclear localization signal (NLS)-conjugated Hic-5 expression vector (NLS-HA-hic-5). NLS-HA-Hic-5 increased the mRNA level of *MMP-13* in human chondrocytes in a dose-dependent manner (Fig. 4A). Similarly, western blot analysis and double-immunofluorescence staining of Hic-5 and MMP-13 showed that MMP-13 protein was increased in NLS-HA-hic-5-expressing chondrocytes compared with untransfected chondrocytes (Fig. 4B,C).

### In vivo knockdown of Hic-5 suppresses the progression of surgically induced OA in rats

**Figure 5:**
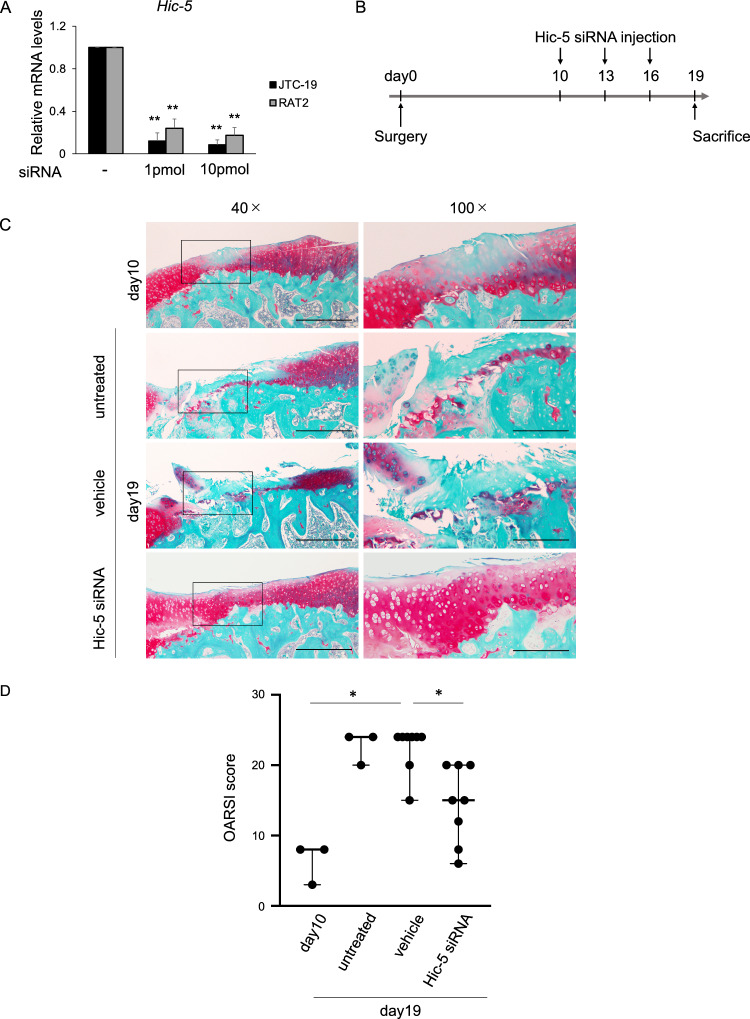
In vivo effect of Hic-5 knockdown on OA in the rat medial meniscectomy (MMx) model. (**A**) mRNA levels of *Hic-5* in rat cell lines transfected with Hic-5 siRNA (n = 5 biological replicates). Values are the mean ± SEM. **P < 0.01, by one-way analysis of variance with Tukey’s test for multiple comparisons. (**B**) Timeline for the surgical procedure of OA induction and treatment by intra-articular injection of siRNA. At 10 days after surgery, rats were sacrificed to verify OA induction (day 10) (n = 3). Hic-5 siRNA (n = 8) or nuclease-free water as the vehicle (n = 8) were administrated to the intra-articular spaces of knee joints at 10, 13 and 16 days after surgery (3 injections in total) and untreated rats were the control (n = 3). (**C**) Representative Safranin-O staining of tibial cartilage in rats subjected to MMx. Right panels show higher magnification views of the boxed areas in left panels. Original magnification × 40 in left panels, Bar = 500 µm; × 100 in right panels, Bar = 200 μm. (**D**) OA Research Society International (OARSI) score of cartilage degradation in the indicated groups. Lines represent the median. *P < 0.05, by the Kruskal–Wallis test, followed by Dunn’s multiple comparisons test.

We evaluated the therapeutic potential of Hic-5 in OA development using a rat surgical model of OA. First, we designed rat Hic-5 siRNA and validated the effect in both JTC-19 and RAT-2 rat cell lines. *Hic-5* expression was remarkably suppressed in Hic-5 siRNA-expressing cells compared with the control (Fig. 5A). Next, Hic-5 siRNA was injected into the intra-articular spaces of rat knee joints every 3 days from day 10 to 19 after medial meniscectomy (MMx) (Fig. 5B).

**Figure 6:**
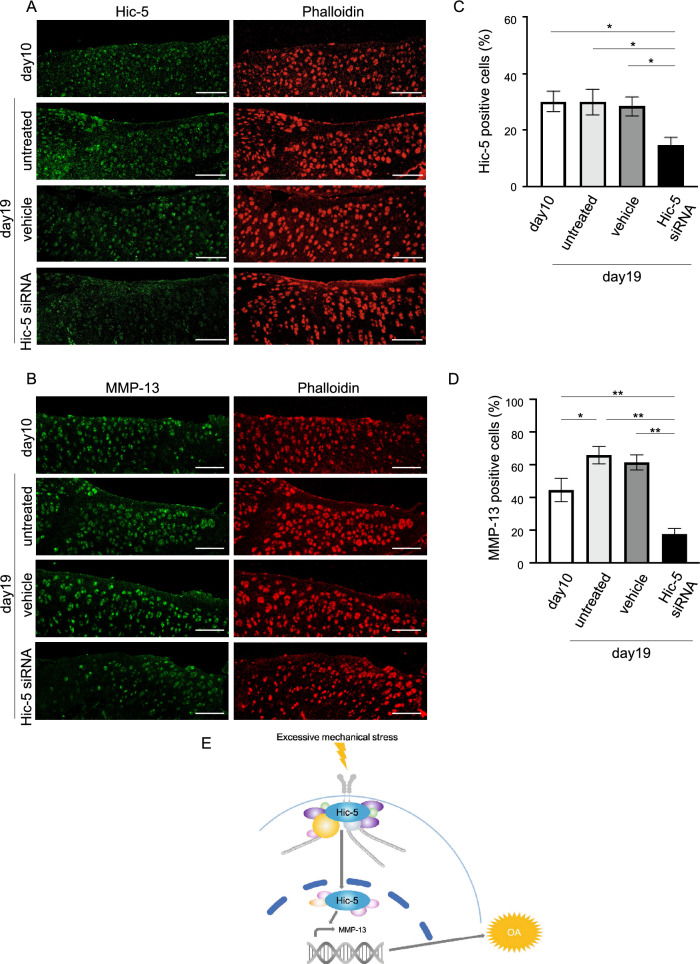
In vivo suppression of MMx-induced protein expression of Hic-5 and MMP-13 by intra-articular injection of Hic-5 siRNA into rats. (**A**,**B**) Representative immunofluorescence of Hic-5 (**A**, green) and MMP-13 (**B**, green) in MMx-operated tibial cartilage with intra-articular injection of Hic-5 siRNA (n = 8) or vehicle (n = 8), or MMx-operated tibial cartilage without treatment (day 10, n = 3 and untreated 19 days after MMx, n = 3). Phalloidin (red) was used to count total cells. Original magnification × 200, Bar = 100 µm. (**C**,**D**) Rates of Hic-5-positive cells (**C**) and MMP-13-positive cells (**D**) in tibial cartilage of rats. Values are the mean ± SEM. *P < 0.05; **P < 0.01, by one-way analysis of variance with Tukey’s multiple comparisons test. (**E**) Schematic model showing that excessive mechanical stress induces MMP-13 expression through Hic-5, which results in OA development.

Histological analysis showed that the formation of OA lesions had already occurred at 10 days postoperatively. At day 19, which was 9 days after the start of siRNA injection, the siRNA-injected rat group showed inhibited OA progression and lower Osteoarthritis Research Society International (OARSI) scores than the vehicle group (Fig. 5C,D and Supplemental Table 2). Additionally, immunohistochemistry confirmed a decrease in Hic-5 and MMP-13 expression in knee cartilage from the siRNA-injected group compared with groups without siRNA injection, including day 10, untreated, and vehicle groups (Fig. 6A–D). Taken together, these results indicated that Hic-5 induced by excessive mechanical stress enhanced OA development by increasing MMP-13 transcription (Fig. 6E).

## Discussion

The current study characterizes Hic-5 as a major regulator of OA development by promoting cartilage degradation. There was a significant increase in Hic-5 expression in human OA cartilage, but not in non-OA cartilage. In vitro experiments showed that excessive mechanical stress induced *Hic-5* and *MMP-13* expression in human chondrocytes, and Hic-5 knockdown suppressed the elevated expression of *MMP-13*, the major ECM-degrading enzyme in OA formation. Additionally, excessive mechanical stress altered the subcellular localization of Hic-5 from focal adhesions to the nucleus, and nuclear accumulation of Hic-5 resulted in the transcriptional regulation of MMP-13. In vivo experiments showed that intra-articular administration of Hic-5 siRNA downregulated MMP-13 expression and had a protective effect against cartilage degradation in OA model rats. Taken together, these results indicated that Hic-5 might be a promising therapeutic target for OA.

Hic-5 is involved in the pathogenesis of various disorders, such as liver fibrosis, pancreatic fibrosis, and colorectal cancer, through its function as a scaffold for multiple cell signals and a regulator of ECM-related gene expression^16–18^. We previously reported that Hic-5 expression was higher in patients with these disorders than healthy controls. In liver and pancreatic fibrosis, Hic-5 acts as a scaffold for the TGF-β/Smad2 pathway and its deficiency significantly attenuates mouse liver and pancreatic fibrosis by a reduction of collagen production, which is the major ECM component in fibrosis^16,17^. Furthermore, we demonstrated that overexpression of Hic-5 using an adenovirus vector in human normal fibroblasts increased the expression of lysyl oxidase (LOX) that increases ECM stiffness and enhances tumor progression^18^. Even more intriguingly, azoxymethane-induced colorectal tumor incidence was suppressed in Hic-5-deficient mice compared with WT mice. In this study, we similarly found that Hic-5 regulated dissolution of ECM rich cartilage as a regulator of MMP-13 and that knockdown of Hic-5 by siRNA resulted in attenuation of surgically induced OA in rats. Additionally, Hic-5 expression was elevated in human OA cartilage. Taken together, Hic-5 may have a role as an ECM regulator in the process of human OA.

This study revealed the novel finding that mechanical stress induced Hic-5 gene expression and translocation in human chondrocytes. Hic-5 is located predominantly in focal adhesions and found in the nucleus under stimulation by ROS in normal human fibroblasts, and by TGF-β in normal human dermal fibroblasts without mechanical stress^15,19^. However, we previously showed that Hic-5 translocated from focal adhesions to actin fibers after mechanical stress in mouse embryonic fibroblasts^11^. These results and the present data differ in terms of Hic-5 translocation, which may be due to the different mechanical stress conditions and different cell types used in the analysis.

In colorectal cancer, nucleus-accumulated Hic-5 induces expression of LOX that catalyzes crosslinking of collagen fibers to increase ECM stiffness^18^. Furthermore, Hic-5 translocates into the nucleus after mechanical stress and is involved in gene expression of matrix-degrading enzymes in chondrocytes. Stimulation to focal adhesion plaques by increased ECM stiffness is consistent with a mechanical stress-loading condition. Taken together, Hic-5 is likely to play a reciprocal role in regulating ECM microenvironmental rigidity by sensing an increase in ECM stiffness, which leads to nuclear translocation and induction of gene expression related to ECM rigidity control. Although Hic-5 gene expression was slightly increased by mechanical stress, MMP-13 expression was significantly suppressed in human chondrocytes. These results imply that the nuclear translocation of Hic-5 rather than increase in its expression induced by mechanical stress is important for OA development.

We have previously reported that Hic-5 regulates c-fos gene expression through AP-1-, Ets-, and Sp1-binding sites^20^. Additionally, it is well known that most of the MMP family have AP-1-binding sites in their promoter regions and a selective c-Fos/AP-1 inhibitor suppresses MMP-13 expression in human chondrocytes. Moreover, intra-articular administration of the inhibitor suppresses MMP-13 expression in a mouse OA model^21^. Therefore, Hic-5 may promote MMP-13 transcription by interacting with AP-1.

Recently, various studies of animal OA models treated by intra-articular injection of small molecule drugs have been performed to explore the potential of novel therapeutic medicines. For example, intra-articular injection of dexamethasone, rebamipide, or statins prevents OA progression in animal models through downregulation of MMP-13 expression^22–27^. Hic-5 has been conventionally considered difficult to inhibit by small molecular drugs because it is an intracellular adaptor protein and has no enzymatic activity. However, inhibition of Hic-5 in vivo has been recently made possible by nucleic acid therapeutics, a new tool capable of selective gene knockdown across plasma membranes. In the present study, intra-articular injection of Hic-5 siRNA suppressed OA progression in rats. Thus, we not only identified Hic-5 as a therapeutic target for OA, but also established that Hic-5 siRNA may be novel therapeutic medicine for OA.

In summary, we demonstrated that Hic-5 regulates cartilage degradation as a transcriptional mediator of MMP-13. Our findings suggest that Hic-5 siRNA has potential therapeutic application, which may be clinically useful in OA.

## Methods

### Immunofluorescence

Formalin-fixed, paraffin-embedded human articular cartilage tissue sections were purchased from ORIGENE (MD, USA). Normal articular cartilages were derived from 3 males aged 36, 52 and 57. 3 OA cartilages were from 2 males aged 73 and 76, and 1 female aged 87. Rat tibial cartilage was fixed in 10% buffered formalin, decalcified in formic acid, embedded in paraffin, and cut into 4-µm-thick sections.

For immunohistochemistry, signals were detected by the CSAII Biotin-free Tyramide Signal Amplification System (Aglient technologies, CA, USA). Sections were incubated with a primary anti-Hic-5 antibody (1:100; 611165, BD Biosciences, NJ, USA) and anti-MMP-13 antibody (1:150; ab39012, Abcam, Cambridge, UK), and counterstained with DAPI or phalloidin. The numbers of Hic-5- and MMP-13-positive cells were quantified by counting immunopositive cells in sagittal sections of the knee joint at × 200 magnification. The percentage of positive cells was determined using BZ-II Analyzer software (Keyence, Osaka, Japan).

For immunocytochemistry, cultured chondrocytes were fixed in 3.7% buffered formalin and blocked with 3% bovine serum albumin (Sigma Aldrich, Taufkirchen, Germany)/phosphate buffered saline (PBS) containing 0.1% Tween-20. Cells were stained with the primary antibody at room temperature for 1 h and then incubated with Alexa Fluor-conjugated secondary antibodies (Invitrogen, MA, USA).

### Chondrocyte culture and exposure to cyclic tensile strain

Normal human articular chondrocytes obtained from a 26-year-old male and 47-year-old male were purchased from Lonza and cultured in chondrocyte basal medium (Lonza, Basel, Switzerland) in accordance with the manufacturer’s instructions. Passage 3–6 human chondrocytes were used for experiments.

Human chondrocytes were plated onto a silicon chamber coated with fibronectin (354008; Corning, NY, USA) at 4 × 10^4^ cells/chamber. Each chamber had a culture surface of 2 × 2 cm. Cyclic tensile strain (0.5 Hz, 10% elongation) was applied using the NS-550 uniaxial stretching system (STREX, Osaka, Japan) in a CO_2_ incubator.

### Transfection of Hic-5 siRNA and plasmid DNA

Human chondrocytes cultured on silicon chambers were transfected for 4 h with Hic-5 siRNA (forward: 5ʹ-GGACCAGUCUGAAGAUAAG-3ʹ; reverse: 5ʹ-CUUAUCUUCAGACUGGUCC-3ʹ, 10 nmol/L) or control siRNA (10 nmol/L; Ambion, TX, USA) using Lipofectamine RNA iMAX (Invitrogen, MA, USA). Then, the cells were washed with PBS and cultured at 37 °C for 20 h. Thereafter, the cells were subjected to cyclic tensile strain. To express a fusion protein carrying a nuclear localization signal (NLS) at the N-terminal of *hic-5*, an NLS *hic-5* expression vector (NLS-HA-hic-5), which has been described previously^20^, was transfected using Lipofectamine 3000 (Invitrogen, MA, USA).

### Real-time quantitative polymerase chain reaction (qPCR)

Total RNA extraction and reverse transcription were performed with a SuperPrep II Cell Lysis RT Kit for qPCR (TOYOBO, Osaka, Japan) following the manufacturer’s instructions. To quantify mRNA expression of Hic-5, MMP-13, MMP-1, MMP-2, MMP-3, and TIMP-1, real-time qPCR analysis was performed using KOD SYBR q PCR Mix (TOYOBO, Osaka, Japan). Primer sequences are listed in Supplementary Table 1. Target gene expression was normalized to GAPDH using the 2^−ΔΔCt^ method.

### Western blotting

Proteins were extracted from human chondrocytes using lysis solution including 2% sodium dodecyl sulfate (SDS). The proteins were fractionated by SDS-polyacrylamide gel electrophoresis, transferred to polyvinylidene difluoride membrane, and detected using anti-MMP-13 (1:1000; Abcam, Cambridge, UK), anti-HA (1:1000; Proteintech, IL, USA), and anti-GAPDH (1:5000; MBL, Tokyo, Japan) antibodies. The band densities were quantified with Densitograph software (ATTO, Tokyo, Japan).

### Rat OA model

Animal experiments were approved by the Animal Care and Use Committee of UNITECH Co. Ltd. and conducted at UNITECH in accordance with the ethical guidelines. All methods were reported in accordance with ARRIVE guidelines (https://arriveguidelines.org). Seven-week-old Slc:Wistar male rats were housed under a 12-h light cycle in a temperature-controlled room for 1 week before surgery was performed. Experimental OA was induced by MMx^28^. Briefly, anesthesia was induced in rats [medetomidine (2 mg/kg bodyweight), midazolam (0.4 mg/kg), butorphanol (5 mg/kg)]. The knees and surrounding areas were shaved. A longitudinal incision was made on the anterior aspect of the right knee. Then, we transected the medial collateral ligament and anterior cruciate ligament of the right knee. Next, the medial meniscus was removed. The knee capsule and subcutaneous tissue were sutured and the skin was closed. Intra-articular treatment was initiated 10 days after surgery. Either Hic-5 siRNA (forward: 5ʹ-GGAUCAUCUAUACAGCACA-3ʹ; reverse: 5′-UGUGCUGUAUAGAUGAUCC-3′, 10 nmol/L) (n = 8) or nuclease-free water (n = 8) as the vehicle with AteloGene Local Use Quick gelation (Koken, Tokyo, Japan) was injected into the intra-articular spaces of rat knee joints in accordance with the manufacturer’s protocol. OA severity was quantified using the OARSI scoring system by UNITECH Co. Ltd^29,30^.

### Statistical analysis

Data normality was assessed by the Shapiro–Wilk normality test. When the distribution was normal, the unpaired *t* test was used to compare two groups of samples and one-way analysis of variance with Tukey’s multiple comparisons test was used to compare data from more than three groups. The Kruskal–Wallis test followed by Dunn’s test was used to compare nonparametric data from multiple groups. All analyses were performed with GraphPad Prism software. Results are reported as the mean ± SEM. *P*-values of less than 0.05 were considered significant.

## Supplementary Information

Supplementary Information.

## Data Availability

All data generated or analyzed during this study are included in this article. Further enquiries can be directed to the corresponding author.
