# Author Correction: Knockdown of mechanosensitive adaptor Hic-5 ameliorates post-traumatic osteoarthritis in rats through repression of MMP-13

**DOI:** 10.1038/s41598-023-36393-w

**Published:** 2023-06-13

**Authors:** Aya Miyauchi, Masahito Noguchi, Xiao‑Feng Lei, Masashi Sakaki, Momoko Kobayashi‑Tanabe, Shogo Haraguchi, Akira Miyazaki, Joo‑ri Kim‑Kaneyama

**Affiliations:** 1 Department of Biochemistry, Showa University School of Medicine, 1‑5‑8 Hatanodai, Shinagawa‑ku, Tokyo, 142‑8555 Japan; 2 Division of Gastroenterology, Department of Medicine, Showa University School of Medicine, 1‑5‑8 Hatanodai, Shinagawa‑ku, Tokyo, 142‑8666 Japan

Correction to: *Scientific Reports* 10.1038/s41598-023-34659-x, published online 08 May 2023

The original version of this Article contained errors in Figures 2 and 4, where panels 2B and 4B respectively did not display correctly. The original Figures 2 and 4 and accompanying legends appear below.

**Figure 2:**
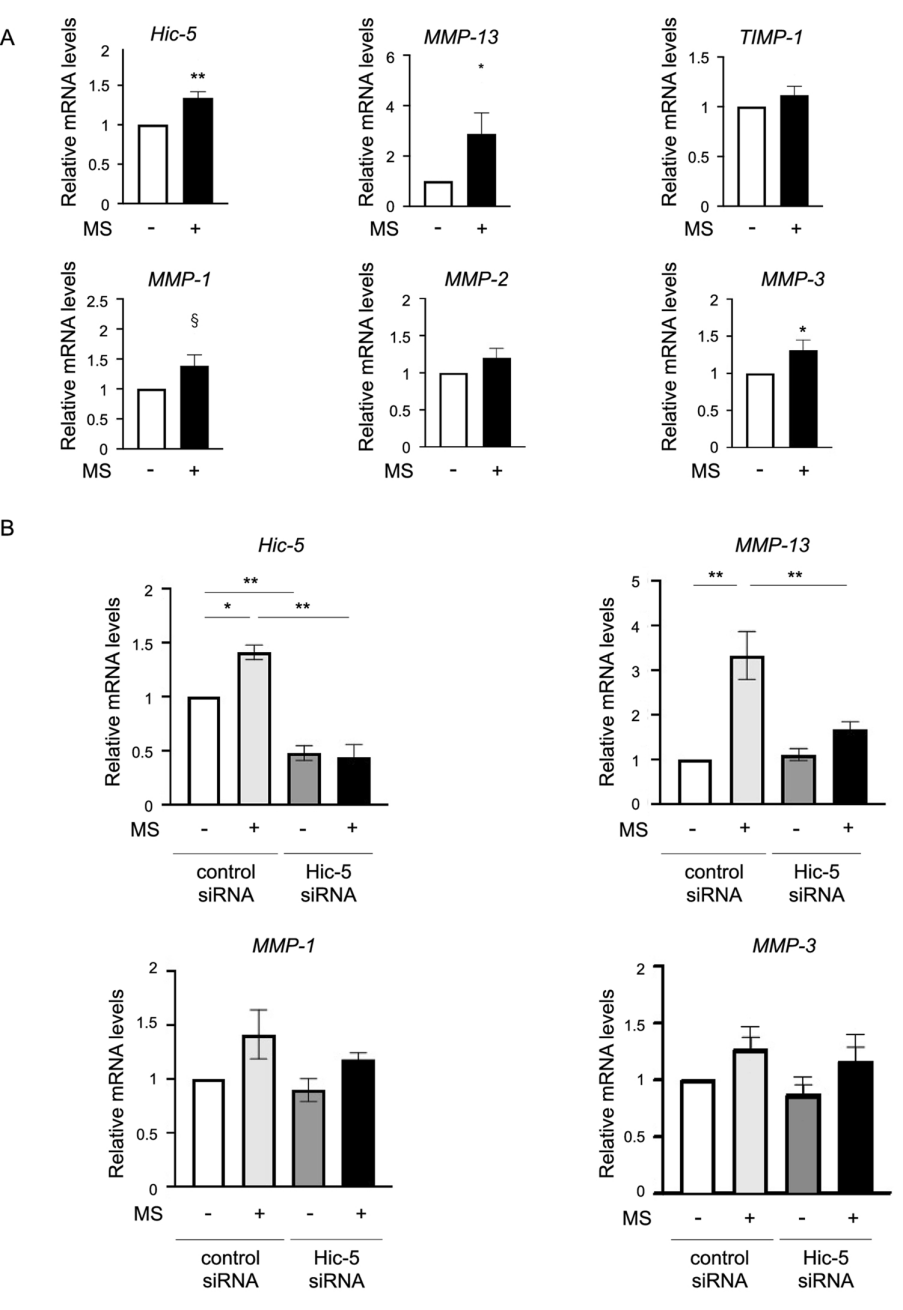
Attenuation of mechanical stress induced-matrix metalloproteinase (MMP-13) expression by Hic-5 knockdown in human chondrocytes. (**A**) mRNA levels of *Hic-5*, *MMPs*, and tissue inhibitor of matrix metalloproteinase-1 (*TIMP-1*) in human chondrocytes exposed to mechanical stress (MS+) for 30 min or untreated (MS−). Cells were collected at 1 h after mechanical stress. (n = 8 biological replicates). (**B**) Changes in gene expression in response to Hic-5 knockdown in human chondrocytes with or without mechanical stress. Human chondrocytes were treated with Hic-5 siRNA (10 nM) or control siRNA (10 nM) for 24 h before stimulation by mechanical stress. (n = 4 biological replicates). Relative levels of mRNA were determined by quantitative reverse transcription-polymerase chain reaction. Values are the mean ± SEM. *P < 0.05; **P < 0.01; ^§^*P* = 0.0572 by the unpaired *t* test in (**A**) or one-way analysis of variance with Tukey’s test for multiple comparisons in (**B**).

**Figure 4:**
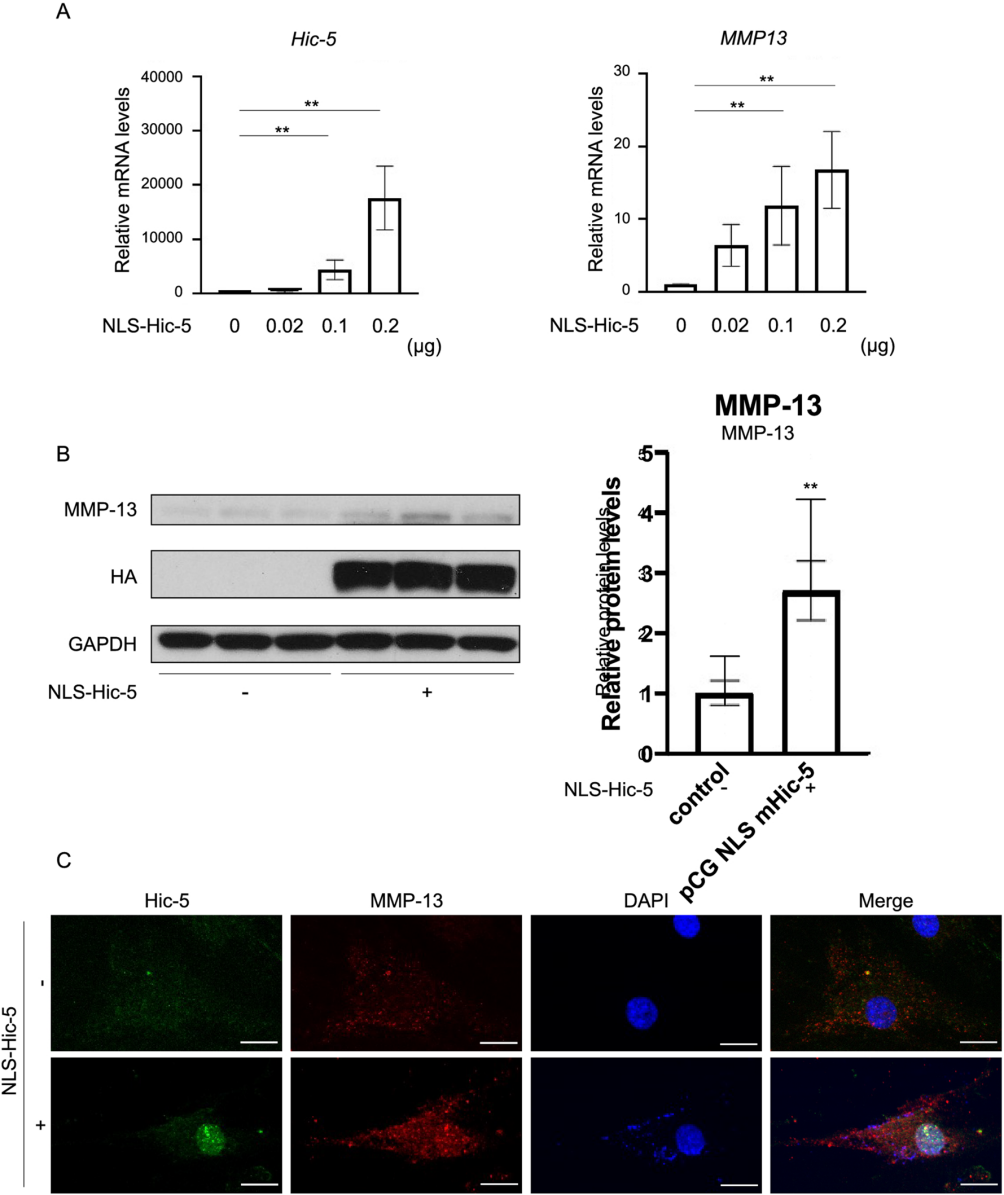
Upregulation of MMP-13 expression by nuclear Hic-5 in human chondrocytes. (**A**) Induction of *MMP-13* in human chondrocytes exogenously expressing Hic-5 tagged with a nuclear localization signal (NLS-HA-Hic-5). Human chondrocytes were transfected with the NLS-HA-Hic-5 expression vector at the concentrations shown in the graph for 24 h. Hic-5 and MMP-13 expression was measured by quantitative polymerase chain reaction (n = 3 biological replicates). (**B**) Western blot of MMP-13 in human chondrocytes transfected with or without 0.2 µg of NLS-Hic-5 (n = 3 biological replicates). Values are the mean ± SEM. **P < 0.01 by the Kruskal–Wallis test, followed by Dunn’s multiple comparisons test in (**A**) or the unpaired *t* test in (**B**). Western blotting images were cropped, and full-length blots are included in Supplementary Fig. 1. (**C**) Double immunofluorescence staining of Hic-5 (green) and MMP-13 (red) in human chondrocytes transfected with or without NLS-HA-Hic-5. Nuclei were counterstained with DAPI (blue). Representative image was selected from 3 biological replicates. Original magnification: × 400. Bar = 50 μm.

The original Article has been corrected.

