# Supplementary material for: Knockdown of mechanosensitive adaptor Hic-5 ameliorates post-traumatic osteoarthritis in rats through repression of MMP-13
Source: Sci Rep. 2023 May 8;13:7446. doi: 10.1038/s41598-023-34659-x (PMC10167244; doi:10.1038/s41598-023-34659-x)
Supplement: Supplementary file 1 — Supplementary Information. [file 41598_2023_34659_MOESM1_ESM.pdf]

透かしを削除する Wondershare PDFelement

Western blot data (full size)

Short exposure

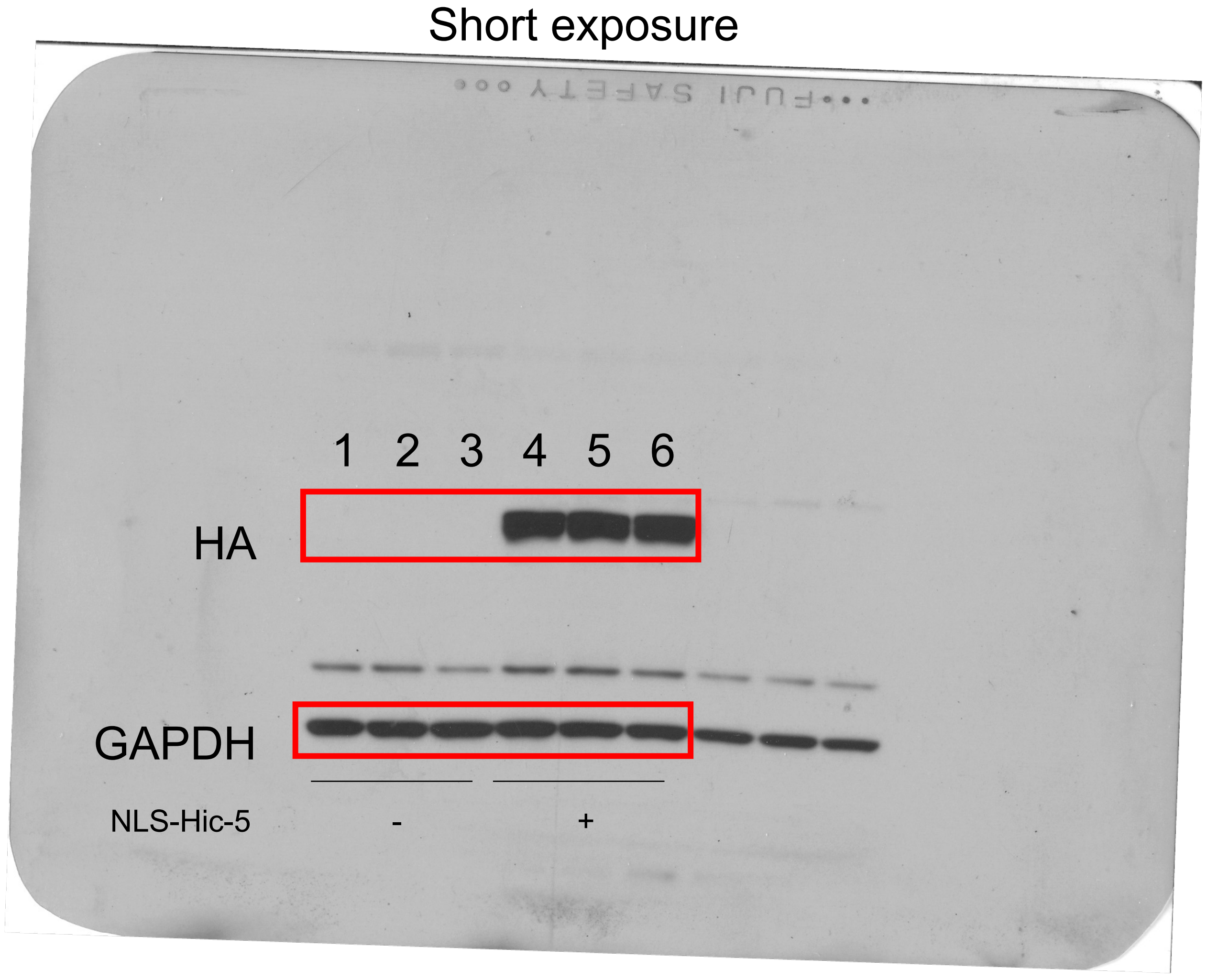

Long exposure

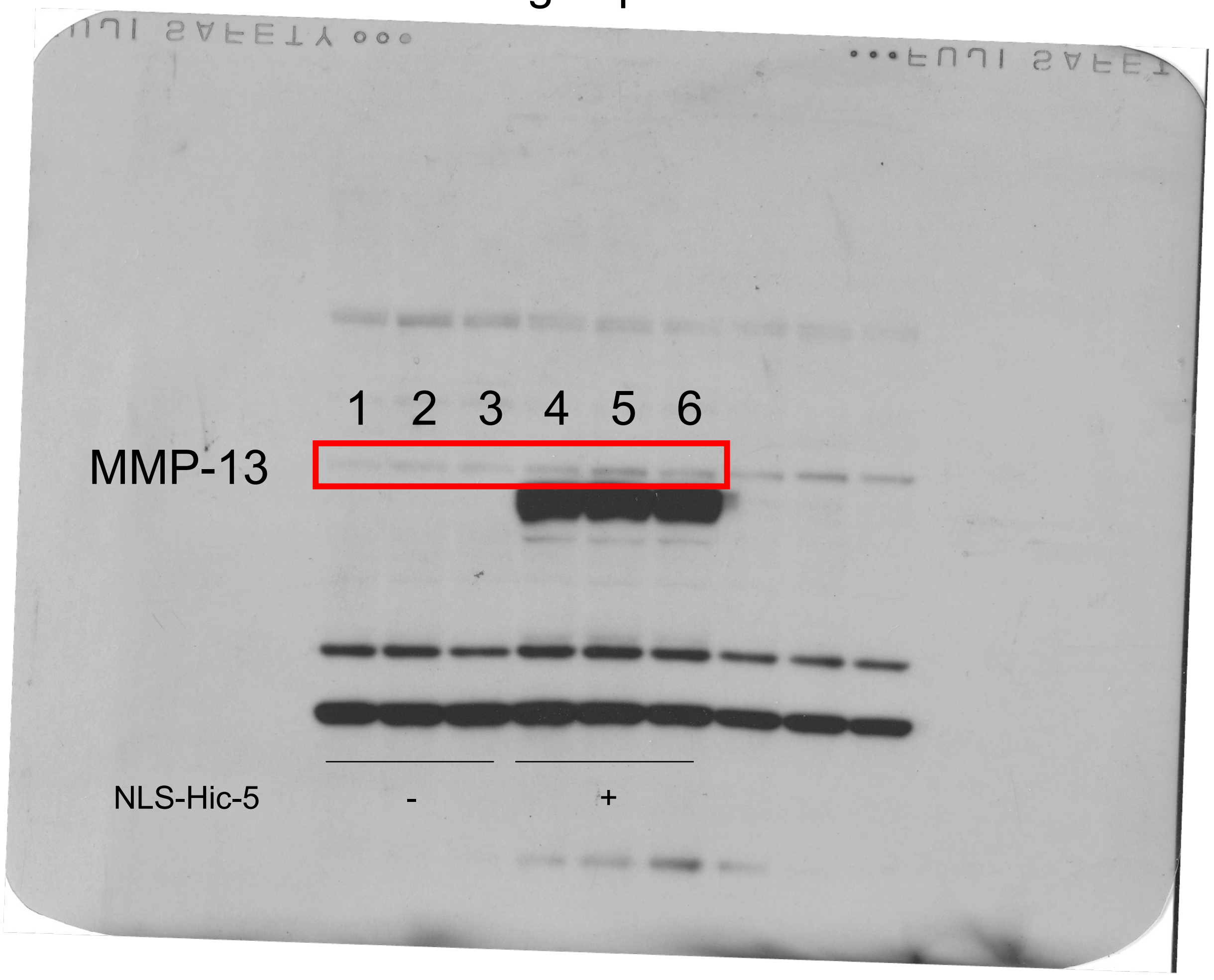

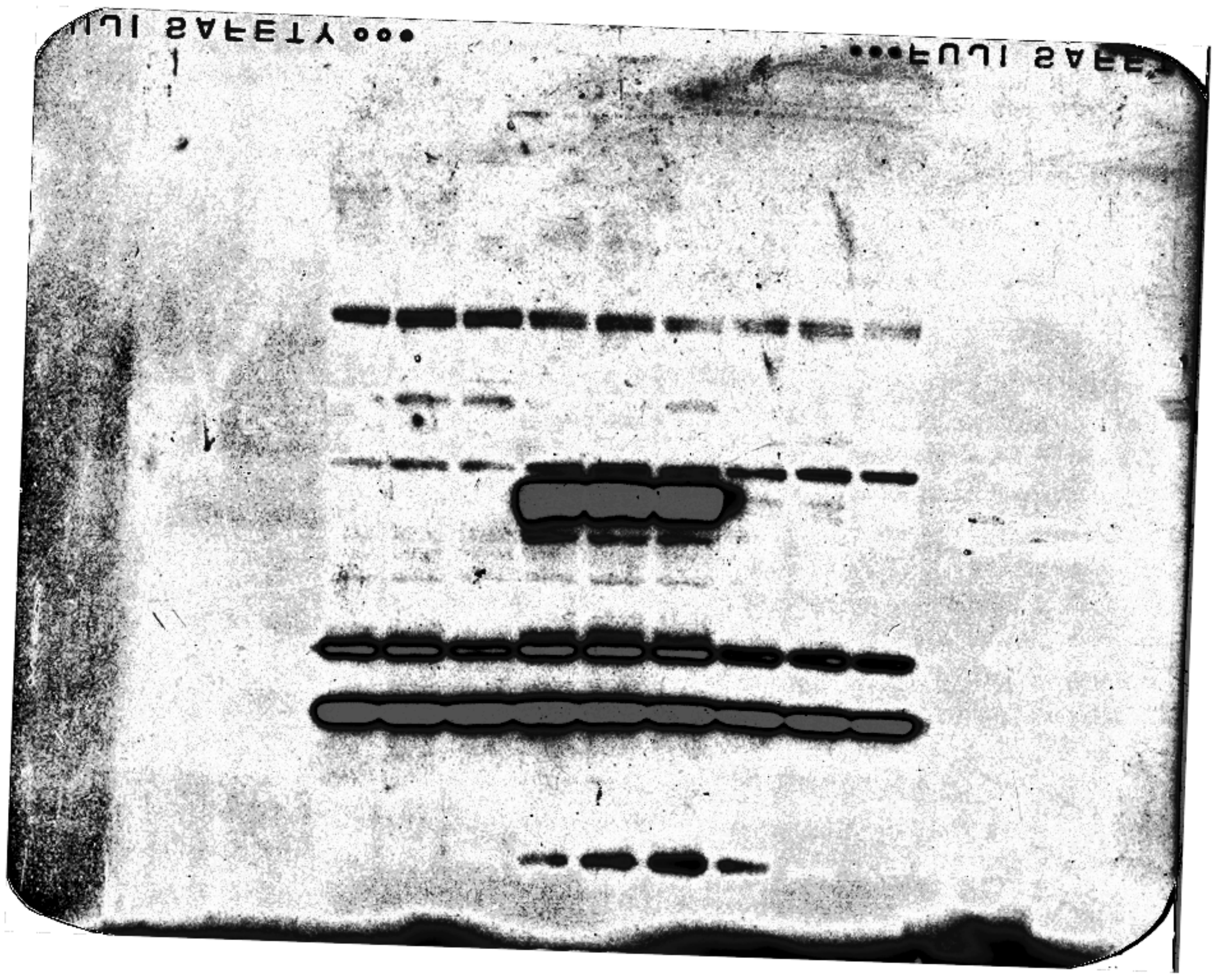

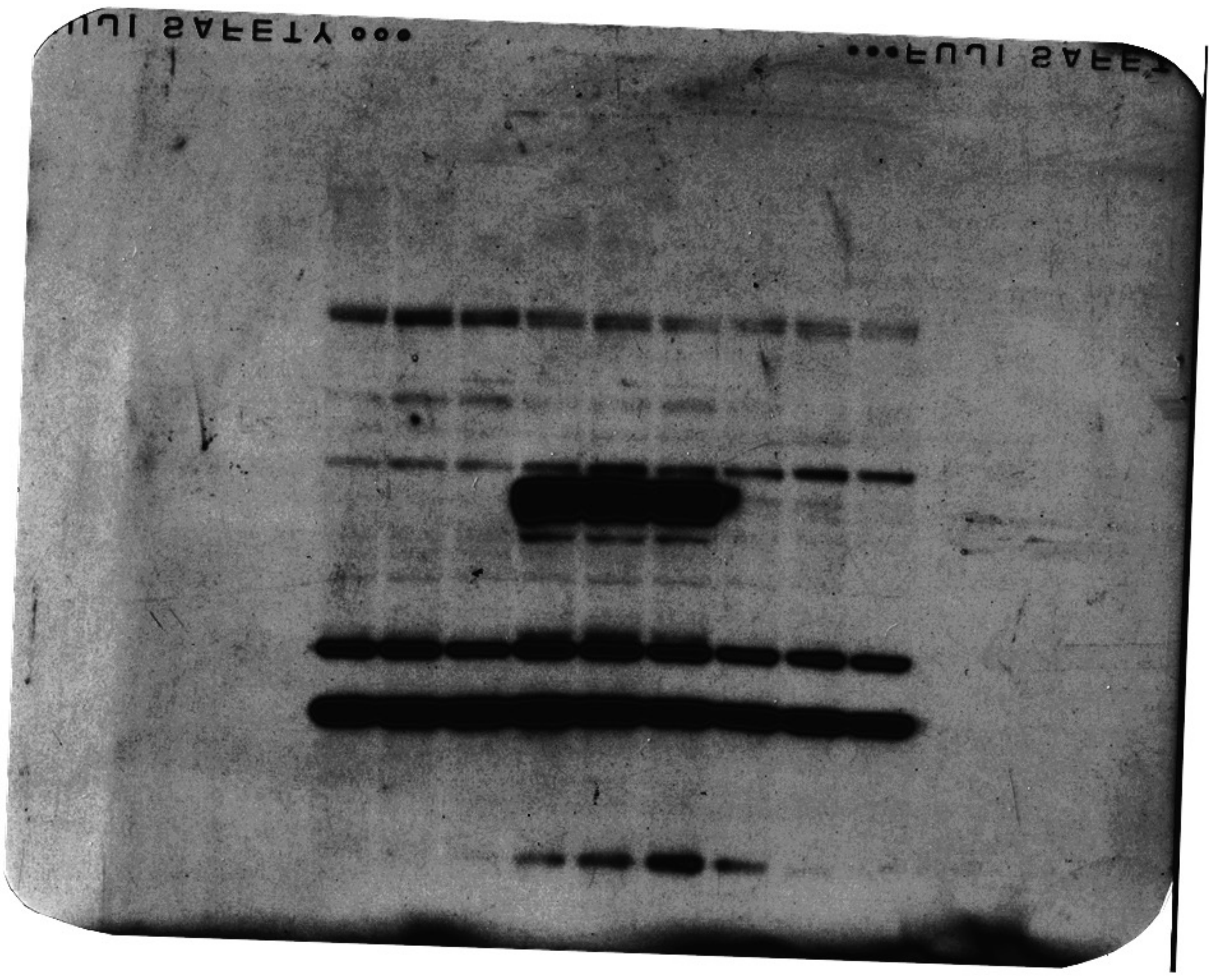

Lane 1, 4 : exp.1
Lane 2, 5 : exp.2
Lane 3, 6 : exp.3

透かしを 削除する Wondershare PDFelement

Supplemental Table 1. Primers for real-time PCR.

Genes	Strand	Primer sequences	Origin
GAPDH	S	5′- GCACCGTCAAGGCTGAGAAC -3′	Human
	AS	5′- TGGTGAAGACGCCAGTGGA -3′	
Hic-5	S	5′- AGTGCTACTTTGAGCGCTTCTC -3′	Human
	AS	5′- GCCGAAGAGCTTCAGGAAGCAAGG -3′	
MMP-13	S	5′- GTCTCTCTATGGTCCAGGAGATGAA -3′	Human
	AS	5′- AGGCGCCAGAAGAATCTGT -3′	
MMP-1	S	5′- CTGTTCAGGGACAGAATGTGCT -3′	Human
	AS	5′- TCGATATGCTTCACAGTTCTAGGG -3′	
MMP-2	S	5′- TGACATCAAGGGCATTCAGGAG -3′	Human
	AS	5′- TCTGAGCGATGCCATCAAATACA -3′	
MMP-3	S	5′- ATTCCATGGAGCCAGGCTTTC -3′	Human
	AS	5′- CATTTGGGTCAAACTCCAACTGTG -3′	
TIMP-1	S	5′- GGATACTTCCACAGGTCCCACAA -3′	Human
	AS	5′- CTGCAGGTAGTGATGTGCAAGAGTC -3′	
GAPDH	S	5′- GGTGAAGGTCGGAGTCAACGGA -3′	Rat
	AS	5′- GAGGGATCTCGCTCCTGGAAGA -3′	
Hic-5	S	5′- CCAGGACAGACCAACAAGGG -3′	Rat
	AS	5′- AAAAGGGAGCCCCATCCTTC -3′	

S, sense; AS, antisense.

透かしを削除する Wondershare PDFelement

Supplemental Table 2. Histopathological changes in the knees of the four studied groups.

	Day 10 (n=3)	untreated (n=3)	vehicle (n=8)	Hic-5 siRNA (n=8)
cartilage matrix loss 0% (mm)	0.398 ± 0.008	1.102 ± 0.142 *	1.152 ± 0.060 †	0.625 ± 0.067 ‡, §
cartilage matrix loss 50% (mm)	0.301 ± 0.035	0.439 ± 0.061	0.529 ± 0.111	0.308 ± 0.107
cartilage matrix loss 100% (mm)	0 ± 0	0.256 ± 0.058	0.233 ± 0.079	0.061 ± 0.030
cartilage degeneration score	6 ± 0.577	8 ± 0.577	7.75 ± 0.590	6.25 ± 0.940
total cartilage degeneration width (mm)	0.772 ± 0.008	1.248 ± 0.185 **	1.180 ± 0.056 ††	0.896 ± 0.068 ‡‡, §§
significant cartilage degeneration width (mm)	0.610 ± 0.070	0.988 ± 0.290	0.808 ± 0.106	0.591 ± 0.092
osteophyte score	2.333 ± 1.528	3.667± 0.5774	4 ± 0	3.714 ± 0.488

* *P*=0.0007 vs Day 10

† *P*<0.0001 vs Day 10

‡ *P*=0.0048 vs untreated

§ *P*<0.0001 vs vehicle

** *P*=0.0302 vs Day 10

†† *P*=0.0245 vs Day 10

‡‡ *P*=0.0591 vs untreated

§§ *P*=0.0357 vs vehicle

Values are the mean ± SEM. Data were analyzed using one-way ANOVA test followed by Tukey's multiple comparisons test.
